# Industrial Masonry Chimney Geometry Analysis: A Total Station Based Evaluation of the Unmanned Aerial System Photogrammetry Approach

**DOI:** 10.3390/s21186265

**Published:** 2021-09-18

**Authors:** Mladen Zrinjski, Antonio Tupek, Đuro Barković, Ante Polović

**Affiliations:** 1 Chair of Instrumental Technique, Faculty of Geodesy, University of Zagreb, Kačićeva 26, 10000 Zagreb, Croatia; mladen.zrinjski@geof.unizg.hr; 2 Chair of Land Surveying, Faculty of Geodesy, University of Zagreb, Kačićeva 26, 10000 Zagreb, Croatia; djuro.barkovic@geof.unizg.hr; 3 GEO-KOM d.o.o., Ban Josip Jelačić Street 87, 47250 Karlovac, Croatia; ante@geo-kom.hr

**Keywords:** masonry chimney, geodetic reference network (GRN), total station (TS), unmanned aerial system (UAS), photogrammetry, point cloud (PC), elliptical regression, polynomial regression, axis inclination

## Abstract

High industrial chimney inclination monitoring and analysis is crucial from a stability point of view because, if not maintained, it can pose a great potential hazard for its surroundings. Various modern approaches of chimneys’ geometrical parameters determination have been proposed and are actively in use. However, little research regarding the applicability of the unmanned aerial system (UAS)-based photogrammetric approach of chimney structural monitoring has been conducted and a comprehensive analysis with validated methods is lacking. Therefore, this research is focused on the determination of geometrical structural parameters of a masonry chimney whereby two independent methods have been applied. Reference values of the chimney geometrical parameters have been determined by precise total station (TS) measurements and, in relation to them, the applicability of the UAS-based photogrammetric approach is evaluated. Methodologically, the reference and validation values of the chimney geometrical parameters have been determined based on double modeling of the chimney structure. Firstly, cross-sectional elliptical regression has been applied to determine the geometrical values of the chimney at predefined above-ground levels (AGLs). Secondly, the spatial chimney axis has been derived by polynomial regression to determine the inclination of the full chimney structure. Lastly, the structural stability of the chimney is validated based on its axis inclination whereby permitted deviations are determined according to the European Standard EN 1996-1-1:2005. Experimental results of our research show that consistently better results are gained by TS-based surveys and, although the determination of the chimney’s geometrical values by the UAS-based approach is certainly possible, great attention must be given to the accuracy of the UAS-generated point cloud (PC) if high accuracy results are needed.

## 1. Introduction

One of the key aspects of the Industrial Revolution period of the 18th and 19th centuries and the construction of many steam-powered factories is the appearance of associated industrial chimneys. Their main function was to evacuate smoke and gases produced by burning coal that was used in almost all industrial facilities. Many factors such as topography, wind, type of industrial factory, nearness to cities, etc. [1,2] influenced the height of the constructed chimney. Therefore, to assure that the surrounding population was not affected by the released waste gases and smoke, chimney heights varied greatly, from several tens to over one hundred meters. Most industrial chimneys of that period are constructed from brick masonry [3] and are conical in shape [3,4,5]. After the Second World War, changes in industrial production and rapid urban expansion led to the closure of many factories whereby their chimneys were most often abandoned and as such rapidly degraded. Moreover, in recent times, many industrial chimneys, due to their great social and historical importance, value, and legacy, have been declared as protected buildings [1,2,3] which, unfortunately, often negatively affected their revitalization. In rare, best-case scenarios where they renewed and maintained. Furthermore, initially constructed on the outskirts of cities, due to urbanization, chimneys can today often be found in densely populated and very active areas of cities. Therefore, if not maintained, they can pose a great potential hazard, because catastrophic structural collapses could cause great material damage and even human casualties. Based on all mentioned factors, the importance and need for analysis of the stability of old industrial chimneys is evident and well explained.

To analyze the stability of the chimney the inclination of the chimney axis is one of the key parameters [1]. In recent times, various approaches of chimney geometrical parameters determination utilizing modern measurement techniques have been proposed. Kocierz and Ortyl [6] investigated the application of modern total stations (TSs) in the determination of the chimney structure inclination and concluded that this approach satisfies the imposed accuracy requirements. In accordance with the findings of [6], Zrinjski et al. [7,8] and Marjetič et al. [9] showed that by utilizing TS measurements, and with a relatively small data set, an accuracy of the determined chimney axis inclination at a level of a few tens of arcseconds or better can be achieved. Furthermore, Zheng et al. [10] proposed a method which combines the basic principles of close-range photogrammetry and traditional engineering surveying and is based on the measurements of the chimney silhouette by means of an imaging station (IS), and thus greatly increases the efficiency of field work. Extensive research in the application of terrestrial laser scanners (TLSs) in deformation analysis of the chimney structure has been conducted [1,11,12,13,14,15,16]. The TLS approach is highly effective in acquiring large data sets, but based on the type of measured chimney, the method is not always applicable. Daliga and Kurałowicz [16] conclude and emphasize that laser scanner measurement, due to surface reflection, should be avoided for stainless steel chimneys. Results obtained on masonry and concrete chimneys suggest TLSs are, for these types of chimneys, suitable for deformation analysis and axis inclination determination [11,13]. However, there is not a definite consensus as to whether the TLS approach is favorable to classical TS measurements [12,14]. Furthermore, several authors show Global Navigation Satellite Systems (GNSS) measurements can effectively be used to determine horizontal displacements, and inclinations of tall industrial chimneys [17,18,19]. An additional significance of the application of GNSS measurements is the possibility of determination of structural dynamic information such as dynamic displacement and vibration frequencies [19,20].

The application of unmanned aerial systems (UASs) in all engineering fields has dramatically increased in the last decade mostly due to the development of Structure from Motion (SfM) algorithms [21]. The method has been proven to be extremely effective in remote surveys of objects of all types. Several studies [22,23,24,25,26], regarding the application of UASs in industrial chimney inspection and documentation, have been published. Harshit et al. [26] researched the application of UASs from a more geometrical point of view and compared the results of UAS and light detection and ranging (LiDAR) surveys of an industrial chimney. The authors concluded that better results, in terms of relative accuracy, were gained with LiDAR data, but emphasize that further research is needed.

A review of the limited relevant scientific literature shows that the application of UASs in industrial chimneys analysis has gravitated more towards visual inspection of structures and that UASs have not yet been extensively applied in the research of structural and geometrical analysis of chimneys. Furthermore, little research concerning the accuracy and applicability of the UAS-based photogrammetric approach of chimney structural monitoring has been conducted. Additionally, a comprehensive comparative analysis with validated methods, e.g., TS measurements, is lacking, and as such a promising field of research is open.

The objectives of this research are to (1) apply two independent methods, i.e., TS and UAS photogrammetry surveys, of structural analysis and geometrical parameters determination of an industrial masonry chimney from a static point of view whereby no dynamics of the chimney body is considered; (2) give a comparison of the gained results with respect to the validated TS-based approach; and (3) to evaluate the applicability of the UAS-based approach in the geometrical analysis of industrial chimneys. In several research papers [7,8,9,12], the TS-based approach in chimney geometry determination has been verified, hence this method will be used to generate the reference data relative to which the UAS results will be evaluated. The structural stability of the chimney is validated based on the inclination of its axis whereby the permitted deviations are determined as defined by the European Standard EN 1996-1-1:2005 [27].

The remainder of this paper is organized as follows. Section 2 elaborates on the study area, i.e., the industrial masonry chimney this research is based upon, gives a general research overview, presents the data acquisition and processing methodologies, and lastly, describes the mathematical methods of chimney geometry determination. Section 3 presents, analyzes, and discusses the gained experimental research results. Finally, Section 4 evaluates the research objectives and concludes the paper.

## 2. Materials and Methods

### 2.1. Study Area

**Figure 1:**
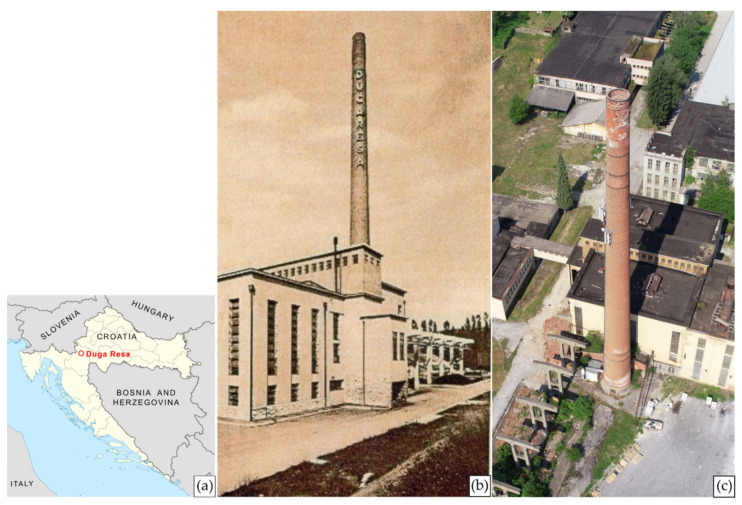
(**a**) Location of the studied industrial chimney with (**b**) its historical depiction [28] and (**c**) a present-day aerial view.

The proposed methodology of chimney structural analysis and inclination determination has been practically applied on an industrial masonry chimney located in the former Duga Resa Cotton Industry in Duga Resa, Croatia (Figure 1).

The masonry chimney was constructed in 1930 in the form of a truncated cone with a bottom and top diameter of approx. 5.5 and 3.0 m. At its construction, the chimney was circa 75 m high, but due to its shortening in 1982, today it is roughly 65 m in height [7,8,28].

**Figure 2:**
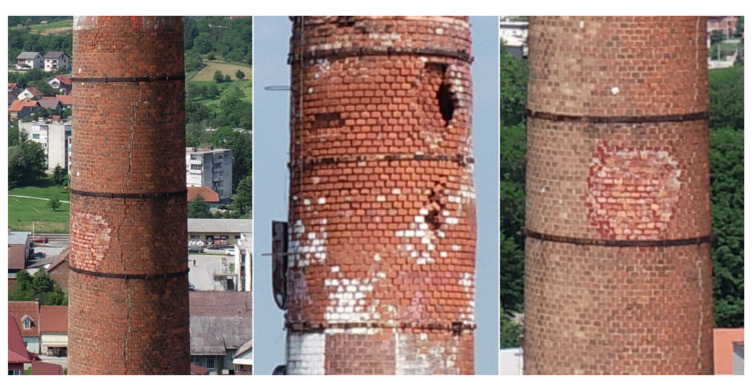
Current condition and structural damage of the studied industrial masonry chimney of the former Duga Resa Cotton Industry in Duga Resa, Croatia.

In the early 1990s, the Duga Resa Cotton Industry suffered significant destruction during the Croatian War of Independence, some of the damage being related to the chimney itself [28]. With the changing social and political conditions of that period, the production slowed more and more every year, until 2015 when the factory completely shut down [29]. However, the chimney has not been in use for nearly 30 years and, due to the lack of maintenance, significantly degraded. Today it is utilized only as a telecommunication antenna carrier. By simple visual analysis of the chimney structure (Figure 2), one can conclude that it may be in an alarming condition. Therefore, based on all stated facts, the urgent need for a geodetic survey and structural analysis of the chimney is crucial and well explained. This additionally contributes to the importance of this research.

### 2.2. General Research Workflow

**Figure 3:**
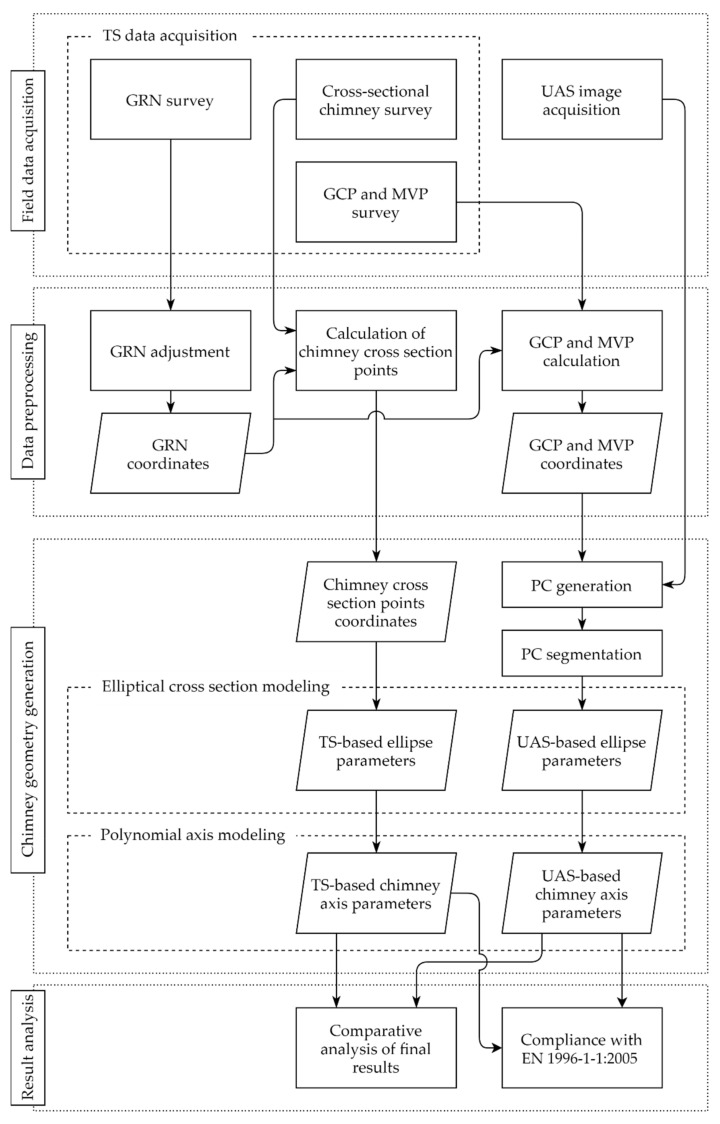
General research workflow; GRN—Geodetic Reference Network; GCP—Ground Control Point; MVP—Model Validation Point; UAS—Unmanned Aerial System; PC—Point Cloud; TS—Total Station.

In this subsection a general overview of the proposed methodology for geometrical structural parameters determination based on a geodetic survey of the industrial masonry chimney of the former Duga Resa Cotton Industry, utilizing TS and UAS photogrammetry approaches, is given. The research workflow (Figure 3) consists of the following four main steps described in detail below:

1. Field data acquisition.

Prior to any measurements of the chimney structure, a geodetic reference network (GRN) is established. It defines the reference coordinate system, i.e., the geodetic datum, for all following measurements and calculations. Chimney geometry reference values are generated utilizing a TS by measuring points on the circumference of elliptical horizontal chimney cross sections by the polar surveying method. Equally, ground control points (GCPs) and model validation points (MVPs), used in the following photogrammetric processing stage, are surveyed. The last part of field survey is the image acquisition of the chimney structure utilizing an unmanned aerial vehicle (UAV).

2. Data preprocessing.

Within data preprocessing, the first step is the least-squares adjustment of the GRN using the Gauss–Markov model (GMM). Coordinates of the network are the framework for calculating (1) points of the chimney surface used to derive reference values of the chimney geometry (cross-sectional point measurement), and (2) GCPs and MVPs used to generate and evaluate a point cloud (PC) of the chimney.

3. Chimney geometry generation.

The central part of the proposed methodology of chimney geometrical parameters determination and UAS-based approach validation is the generation of its geometry. The chimney geometry is determined twice based on two independently generated data sets from the TS survey (TS data set) and the UAS photogrammetric survey (UAS data set). Centers of the chimney and its cross-sectional dimensions are generated based on horizontal elliptical cross-sectional modeling. Furthermore, based on the predetermined *n* cross section centers, the chimney axis is modeled by using an appropriate spatial function, i.e., a curve. The complete modeling is conducted utilizing regression analysis.

4. Result analysis.

The final methodological step includes a comparative analysis of the results gained by two independent methods and evaluation of the applicability of the UAS photogrammetric approach. Furthermore, inclination of the chimney axis is compared with the maximal allowed imperfections defined by the European Standard EN 1996-1-1:2005 [27].

### 2.3. Field Data Acquisition

**Figure 4:**
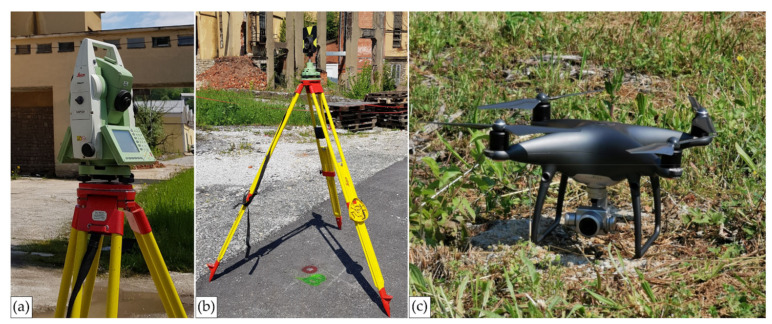
Geodetic sensors and accessories used in this research; (**a**) total station (TS) Leica Geosystems TCRP1201+ R400; (**b**) survey accessories: tripod (Leica Geosystems GST20); tribrach (Leica Geosystems GDF322); reflector carrier (Leica Geosystems GRT144); reflector (Leica Geosystems GPR121); (**c**) unmanned aerial vehicle (UAV) DJI Phantom 4 Pro.

All field data have been acquired based on four geodetic surveys utilizing a total station Leica Geosystems TCRP1201+ R400 (Figure 4a) for all TS-based measurements, and a UAV DJI Phantom 4 Pro for UAS image acquisition (Figure 4c). Detailed information on the filed data acquisition is summarized in Table 1.

#### 2.3.1. GRN Survey

**Figure 5:**
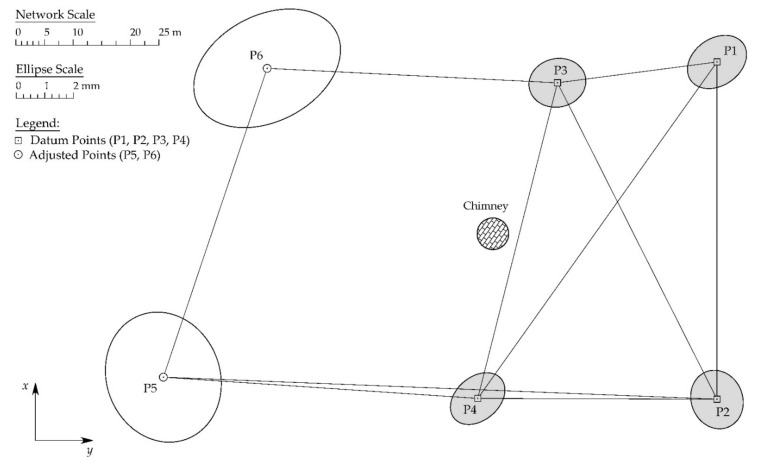
The geodetic reference network (GRN) with corresponding absolute confidence point error ellipses defined at a confidence level of 95%. The network datum is defined from four points of the braced quadrilateral (shaded ellipses).

To gain a well-defined and homogenous reference frame for the planned TS and UAS surveys, a geodetic reference network has been established. The network (Figure 5) was defined as a classical 3D braced quadrilateral (P1–P4) with two additional points around the chimney (P5 and P6). All points have been stabilized by steel geodetic bolts at ground level. All network observations (horizontal directions, zenith angles, and slope distances) have been measured by conventional terrestrial total station (TS) methods whereby the force centering method has been applied. Figure 4b depicts the original Leica Geosystems surveying accessories used during network survey. The geometry of the network is not ideal but is optimal considering all field restrictions one can expect in an industrial facility. During all network measurements, ambient atmospheric parameters (air temperature, air pressure, and air humidity) have been continuously recorded by a handheld meteorological device Lufft XA1000 with a precise temperature and humidity probe Lufft 8130.TFF (G. Lufft Mess- und Regeltechnik GmbH, Fellbach, Germany).

#### 2.3.2. TS Reference Data

To evaluate the chimney geometry results gained with the UAS photogrammetric survey, referent measurements of the chimney structure have been conducted. All referent measurements have been acquired utilizing the Leica Geosystems TCRP1201+ R400 total station (Figure 4a). Relevant technical specifications of the used total station can be found in the official Leica TPS1200 User Manual [30]. However, the instrument’s angle and distance measuring precisions have been evaluated according to International Standards ISO 17123-3:2001 [31] and ISO 17123-4:2012 [32] before any field measurement have been conducted. The results showed that the horizontal and vertical angle and distance measurement precisions fulfill the specifications declared by the manufacturer.

To obtain reference values of the chimney geometry, a cross-sectional survey of the chimney structure has been conducted utilizing the polar surveying method by measuring points on the circumference of elliptical horizontal chimney cross sections. Thereby, slope distances have been measured by the instrument’s reflectorless electronic distance measurement (EDM) module. Although, theoretically, horizontal cross sections of a truncated cone chimney are circles, in this research cross sections have been modeled as ellipses so that any possible construction flaws and/or deformations of the chimney body to a lesser extent affect the determination of the cross-section center and geometry.

The survey of the chimney structure has been conducted from points P1, P4, and P5 of the preestablished GRN (Figure 5). In total, 14 cross sections, vertically spaced apart approximately 5 m, have been measured with a total of 594 points. The vertical cross-section spacing has been arbitrarily defined under the assumption that, at this value, the chimney inclination can be observed between two neighboring cross-sections. To avoid laser beams with small angles of incident and related errors, no points have been measured near the edge of the chimney’s cross-section.

#### 2.3.3. UAS Image Acquisition

The UAS imagery has been acquired utilizing a DJI Phantom 4 Pro multirotor UAV (SZ DJI Technology Co. Ltd., Shenzhen, China) equipped with a 20-megapixel DJI FC6310 integrated on-board CMOS visible camera (Figure 4c). Relevant technical specification can be found on the official DJI webpage [33]. The UAS flight operations have been conducted in two sessions. The first horizontal flight was planned with 85% endlap and 85% sidelap at an altitude of 80 m, resulting in an approximate ground sample distance (GSD) value of 2 cm. The second flight concentrated on the chimney and consisted of multiple vertical flights around it. Mission planning was conducted in 3DSurvey Pilot. A total of 539 images with a resolution of 5472 × 3648 pixels from a vertical and horizontal camera view have been acquired.

An essential part of UAS photogrammetry survey is the placement and survey of GCPs and MVPs prior to image acquisition. Ideally, they are widely and uniformly spatially distributed throughout the whole area of survey. The role of GCPs is crucial as they serve to georeferenced the model in the SfM algorithm. MVPs, commonly known as ground check points, are used to objectively evaluate the accuracy of the georeferenced model as they are not used in the camera alignment procedure [34]. In the case of an industrial chimney UAS survey, the placement and signalization of artificial markers on the chimney’s body is practically difficult to realize solely due to the height of the chimney. Therefore, in this research, well-defined natural characteristic features on the chimney surface have been used as GCPs and MVPs. The drawback is the fact that it is very difficult to fulfill the general criteria on the uniform spatial distribution of GCPs and MVPs. In total, 15 GCPs (of which 9 are on the chimney) and 4 MVPs (all of which are on the chimney) have been identified. The positions of these points have been measured utilizing the Leica Geosystems TCRP1201+ R400 total station in the reference coordinate system defined by the preestablished GRN.

### 2.4. Methods

This subsection describes the applied GRN adjustment procedures, TS data processing, UAS imagery processing, the method of cross-sectional ellipse modeling, and methods of chimney axis modeling. For more research examples on the cross-sectional approach of high industrial chimney axis inclination modelling and analysis, an interested reader is referred to [1,13,15,16,26].

#### 2.4.1. GRN Adjustment and TS Data Processing

Prior to GRN adjustment, measured slope distances were corrected for meteorological and atmospheric parameters [35,36]. The reference coordinate system of the established network has been defined as a local 3D coordinate system whereby the network datum has been defined from four points of the braced quadrilateral part of the GRN depicted on Figure 5 by shaded ellipses. This approach leads to the definition of the partial minimum trace datum whereby the partial norm of the square cofactor matrix of unknown parameters attains a minimum (p. 25, [37]). Due to the inevitable and undesirable weak geometric configuration of the established network, i.e., points P5 and P6 are not connected by diagonal measurements from P3 and P4, points P5 and P6 are not used as datum points. Reasoning that the weak network configuration would negatively affect the accuracy and quality of the whole network. Coordinates of all network points have been determined by a least-squares adjustment using the Gauss–Markov model (GMM). For more information on network adjustment procedures and datum definitions, an interested reader is referred to [37,38,39].

Coordinates of points on the circumference of elliptical horizontal chimney cross sections, GCPs, and MVPs, all measured by the polar method, have been computed by well-known geodetic formulas. The precision of all points, in the form of standard deviation of coordinates, has been quantified by applying the error propagation law [38].

#### 2.4.2. UAS Imagery Processing

Full photogrammetric data processing of the acquired images and the generation of a 3D chimney point cloud (PC) was conducted utilizing Agisoft Metashape Professional v1.6.3 (Agisoft LLC, St. Petersburg, Russia) and its built-in SfM algorithm [40]. The coordinate system was assigned as a local coordinate system as defined by GCPs. The acquired aerial images have been georeferenced during the image alignment process using the defined GCPs whereby the image tie points have been generated at full image resolution. After the alignment procedure, a dense PC is generated wherein no downsizing of the images has been conducted, i.e., the *ultra-high* processing parameter was selected. The elaborated image processing resulted in a PC of ≈2 million points. The photogrammetric processing was performed utilizing a desktop computer equipped with Intel^®^ Core™ i7-8700 CPU, Nvidia GeForce GTX 1660 GPU, and 24 GB RAM in the Windows 10 OS.

#### 2.4.3. Cross-Sectional Ellipse Modeling

To determine the cross-sectional centers and dimensions of the industrial masonry chimney regression analysis was conducted whereby individual chimney cross sections have been modeled with ellipses so that any construction flaws and/or deformations of the chimney body with less impact affect the obtained results. Since two independent data sets are available, i.e., the TS data and UAS data sets, the modeling was conducted twice using identical mathematical basis. The modeling of TS-based data is straightforward since points have originally been measured only at individual cross sections, that is above ground levels (AGLs). However, since the final product of photogrammetric processing is a PC of the full chimney structure, segmentation along the vertical is necessary. Segmentation of the full chimney PC was conducted in CloudCompare v2.11.3 [41] whereby the AGL of the cross section to be segmented is defined by the AGL of the equal TS-based cross section. Furthermore, knowing the approximate radius of the chimney at the bottom and top and its height it can easily be calculated that the radius gradient is approx. −1.5 cm per 1 m of chimney height. Therefore, to gain correct results, during segmentation a segment height of 5 cm along the vertical was chosen. Thus, the influence of the chimney’s vertical radial gradient on the ellipse modeling is below the 1 mm level and as such is not considered. Since in both approaches identical coordinate systems have been realized, a direct comparison of the gained results is possible.

Due to measurements errors and chimney imperfections, all points on one individual cross section will not define one unique ellipse. Hence, to define the cross-sectional geometry of the chimney, nonlinear elliptical regression analysis is required. The topic of least-squares ellipse fitting has been extensively researched in the last several decades. An interested reader is referred to [42,43,44,45,46]. Generally, least-squares fit on any geometrical feature to a set of given points is based on the minimization of the squares sum of an error distance, i.e., error-to-fit values [43].

In this research, the condition of minimizing the squares sum of error distances ${\bar{\varepsilon }}_{i}$, based on the fundamental definition of an ellipse, is set and a fundamental nonlinear functional model is defined:

(1) $${\bar{\varepsilon }}_{i}=\sqrt{{\left(y_{i}-{\bar{y}}_{F1}\right)}^{2}+{\left(x_{i}-{\bar{x}}_{F1}\right)}^{2}}+\sqrt{{\left(y_{i}-{\bar{y}}_{F2}\right)}^{2}+{\left(x_{i}-{\bar{x}}_{F2}\right)}^{2}}-2\bar{a},\;i=1,2,\ldots ,m,$$

where $\left(y_{i},x_{i}\right)$ are coordinates of the *i*-th point, $\left({\bar{y}}_{F1},{\bar{x}}_{F1}\right)$ are the adjusted coordinates of the first focal point of the ellipse, $\left({\bar{y}}_{F2},{\bar{x}}_{F2}\right)$ are the adjusted coordinates of the second focal point of the ellipse, $\bar{a}$ is the adjusted semi-major axis of the ellipse, and m is the total number of points to which the ellipse is fitted. By determining the coordinates of the ellipse foci and its semi-major axis, the ellipse is uniquely defined. Other ellipse parameters, i.e., coordinates of its center $\left({\bar{y}}_{C},{\bar{x}}_{C}\right)$ and semi-minor axis $\bar{b}$, can be calculated based on generally known ellipse geometry equations.

To solve the stated nonlinear least-squares problem, the Gauss–Newton method [42,43,44,47,48] is applied. The unknowns of the functional model are determined iteratively. Initial guesses of the unknowns are generated, and by using Taylor’s Theorem the functional model of Equation (1) is linearized. The Jacobian matrix is populated from partial derivatives of Equation (1). Employment of the fundamental least-squares principle leads to the formation of the normal equation system, the solution of which determines the unknown ellipse parameters, i.e., the coordinates of the ellipse foci and its semi-major axis. A next iteration of the adjustment is conducted until the solution converges, thereby the initial values are defined as the results of the previous adjustment iteration. The stochastic model of adjustment is defined by the corresponding diagonal weight matrix whereby individual weights are defined based on the known associated standard deviations of horizontal coordinates of all points of the cross section under adjustment. The weighted least-squares ellipse-fitting algorithm is herein briefly described; for more detailed explanation refer to [42,43,44].

The AGLs of individual cross sections, i.e., the heights of the fitted ellipses ${\bar{H}}_{C}$, are determined independently from the previously elaborated ellipse-fitting algorithm. The cross section’s AGL is determined as a weighted mean based on the heights and associated standard deviation of all points of that cross section.

#### 2.4.4. Chimney Axis Modeling

Using nonlinear elliptical regression analysis, the cross-sectional geometry of the masonry chimney at *n* individual AGLs is determined whereby the key parameters for further analysis are the center coordinates of every individual cross section $\left({\bar{y}}_{Cj},{\bar{x}}_{Cj},{\bar{H}}_{Cj}\right)$ for *j* = 1, 2, …, *n*. Since two independent sets of *n* cross-sectional centers are available, i.e., the TS and the UAS data sets, the modeling of the chimney axis is conducted twice while using an identical mathematical basis. From *n* individual cross sections, the first, i.e., the lowest cross section (*j* = 1), is defined as the reference cross section, and the coordinates of its center define the referent vertical relative to which the chimney axis is analyzed. Again, due to chimney imperfections and measurement errors, centers of all cross sections will not define one unique spatial curve. Therefore, to mathematically define the chimney axis, least-squares fitting is necessary. The broad topic of curve fitting is well researched and herein only a brief formulation of the defined functional models is given. An interested reader is referred to [49,50,51,52,53,54,55,56].

In this research, the geometry of the masonry chimney structure, i.e., the parameters of its axis have been mathematically determined by polynomial regression [50,51,52,53,54] wherein the spatial chimney axis has been fitted by a parametric curve represented by a vector-valued polynomial function of degree *k*. Three polynomial functions (*k* = 1, 2, and 3) in 3D have been considered and evaluated:

1. Line (1st degree polynomial—linear function, *k* = 1);
2. Quadratic curve (2nd degree polynomial—quadratic function, *k* = 2);
3. Cubic curve (3rd degree polynomial—cubic function, *k* = 3).

The basic intention of any 3D polynomial regression is to find a least-squares best fit spatial curve $f\left(u\right)$ to a set of *l* given points in ${\mathbb{R}}^{3}$ by minimizing the residual sum of squares (RSS), i.e., error distances, of the points from the curve:

(2) $$\mathrm{RSS}=\sum_{p=\;1}^{l}\omega_{\;p}{\left\|f\left(u_{p}\right)-P_{p}\right\|}^{\;2},\;p=1,2,\ldots ,l,$$

where $f\left(u_{p}\right)={\left[f_{x}\left(u_{p}\right),f_{y}\left(u_{p}\right),f_{z}\left(u_{p}\right)\right]}^{T}$ is the parametric curve function at parameter value $u_{p}$, $P_{p}={\left[x_{p},y_{p},z_{p}\right]}^{T}\in {\mathbb{R}}^{3}$ is a point in 3D Euclidean space the parameter value $u_{p}$ corresponds to, and $\omega_{\;p}$ represents the squared residual weight of the least-squares adjustment.

The mathematical regression model for the determination of the chimney axis by the cubic curve reads:

(3) $$r\left(t_{j}\right)=\left\{\begin{array}{rrrrrrrrr} {\hat{y}}_{Cj} & = & {\bar{y}}_{C1} & + & {\bar{a}}_{y}t_{j} & + & {\bar{b}}_{y}t_{j}^{2} & + & {\bar{c}}_{y}t_{j}^{3}\\ {\hat{x}}_{Cj} & = & {\bar{x}}_{C1} & + & {\bar{a}}_{x}t_{j} & + & {\bar{b}}_{x}t_{j}^{2} & + & {\bar{c}}_{x}t_{j}^{3}\\ {\hat{H}}_{Cj} & = & {\bar{H}}_{C1} & + & {\bar{a}}_{H}t_{j} & + & {\bar{b}}_{H}t_{j}^{2} & + & {\bar{c}}_{H}t_{j}^{3} \end{array}\right.,\;j=1,2,\ldots ,n,$$

where $\left({\hat{y}}_{Cj},{\hat{x}}_{Cj},{\hat{H}}_{Cj}\right)$ are adjusted coordinates of the chimney axis at the *j*-th cross section, $\left({\bar{y}}_{C1},{\bar{x}}_{C1},{\bar{H}}_{C1}\right)$ are coordinates of the reference cross section (*j* = 1), ${\bar{a}}_{y},{\bar{a}}_{x},{\bar{a}}_{H}$ are adjusted linear polynomial function coefficients, ${\bar{b}}_{y},{\bar{b}}_{x},{\bar{b}}_{H}$ are adjusted quadratic polynomial function coefficients, ${\bar{c}}_{y},{\bar{c}}_{x},{\bar{c}}_{H}$ are adjusted cubic polynomial function coefficients, and $t_{j}$ is the curve parameter associated with every cross section center. The dimensionless parameter $t_{j}$ of the corresponding *j*-th cross section is defined by a simple length parametrization based on the center coordinates of that cross section $\left({\bar{y}}_{Cj},{\bar{x}}_{Cj},{\bar{H}}_{Cj}\right)$ and the center coordinates of the reference cross sections $\left({\bar{y}}_{C1},{\bar{x}}_{C1},{\bar{H}}_{C1}\right)$. The parameters $t_{j}$ $\left(j=1,2,\ldots ,n\right)$ are independent variables within the least-squares curve fitting. Since the 3rd degree polynomial function includes the 1st and 2nd degree polynomials, the regression models of the 3D line and 3D quadratic curve fitting are derived from the cubic regression model of Equation (3) by omitting the cubic and quadratic elements. Therefore, the linear and quadratic models are not explicitly stated.

The regression procedure is conducted using the well-known weighted least-squares method under the Gauss–Markov model [55,56]. It is emphasized that, although quadratic and cubic curves are nonlinear fits to the given data points, the regression model is a linear model because it is linear in the unknown parameters, i.e., polynomial function coefficients. By solving the normal equation system, the adjusted unknown parameters, i.e., the polynomial function parameters, are computed. The stochastic model of the adjustment is defined by the corresponding diagonal weight matrix whereby the weight of every individual observation, i.e., point coordinate, has been determined based on the associated standard deviation of that coordinate, according to the theoretical definition of weight.

After the determination of the spatial chimney axis by a vector-valued polynomial function of degree *k* (*k* = 1, 2, and 3) the unit tangent vector $G\left(t\right)$ of the parametric curve $r\left(t\right)$ at parameter value *t* is given by the following equation:

(4) $$G\left(t\right)=\frac{r^{\prime }\left(t\right)}{\left\|r^{\prime }\left(t\right)\right\|}.$$

Finally, the inclination of the chimney φ at any point defined by *t* can be calculated as the angle between the vertical *H*-axis of the coordinate system and the unit tangent vector $G\left(t\right)$:

(5) $$\cos\varphi =\frac{g_{3}}{\sqrt{g_{1}^{2}+g_{2}^{2}+g_{3}^{2}}},$$

where $g_{1},\;g_{2},\;g_{3}$ are components of the unit tangent vector $G\left(t\right)$.

To obtain an insight into the significance of the determined chimney inclination to the vertical at point *t*, it is compared with the maximal allowed imperfections of masonry structures as defined by the European Standard EN 1996-1-1:2005 [27]:

(6) $$\varphi_{\max}\left[\mathrm{rad}\right]=\frac{1}{100\sqrt{H}}.$$

## 3. Results and Discussion

According to the elaborated methodology of GRN establishment and least-squares adjustment, final coordinates of all network points with corresponding quality criteria parameters, are determined and presented in Table 2. Full visualization of the established GRN is given on Figure 5. The network’s accuracy distribution is visualized by absolute confidence point error ellipses defined at a confidence level of 95%.

To evaluate the global measure of accuracy of the established GRN, the standard deviation of point position, according to Mittermayer [57], is calculated and amounts to:

(7) $$s_{M}=0.61\;\mathrm{mm}.$$

Based on the accuracy assessment parameters of the GRN, i.e., standard deviations of the network coordinates, parameters of the absolute error ellipsoids and the global measure of accuracy, a high degree of network precision and accuracy is evident. The accuracy distribution of individual network points is primarily determined by the network configuration and observations scheme, which is evident from the orientation of the error ellipses. It all implies the absence of gross errors and systematic influences in network observations. To conclude, the network adjustment result is evaluated as high quality and satisfactory.

**Figure 6:**
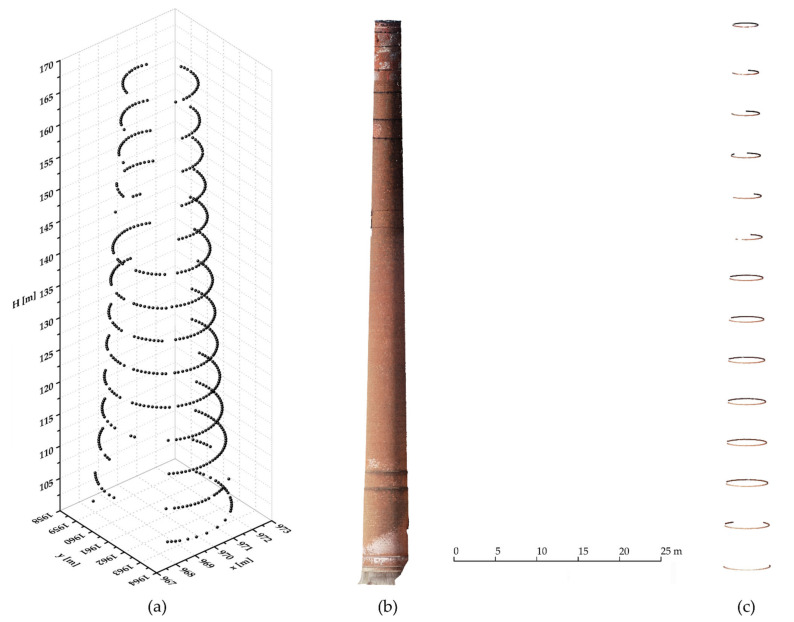
Total Station (TS) and Unmanned Aerial System (UAS) data processing results; (**a**) 3D visualization of the TS-based data set of the chimney; (**b**) chimney full point cloud (PC) generated by photogrammetric processing; (**c**) chimney PC cross-sectional segmentation results.

Consistent with the elaborated research workflow (Figure 3), field survey data has been processed according to the previously described methods (Section 2.4) which led to the generation of the reference data set acquired by TS measurements, and the UAS data set based on photogrammetric processing. 3D coordinates of all 594 points distributed along 14 chimney cross sections with their corresponding accuracy values (standard deviations) have been determined. A visualization of the gained TS-based results is given on Figure 6a. The elaborated photogrammetric processing method of the acquired imagery resulted in a full chimney point cloud. However, due to the structural damage of the chimney, i.e., the holes on the chimney surface (Figure 2), and the telecommunication antennas it carries, the generated point cloud was affected with a certain level of noise. Therefore, prior to further processing, the cloud has been manually cleaned whereby all outlier points, that do not relate to the chimney surface, have been removed. The resulting chimney cleaned PC consisting of ≈2 million points is depicted on Figure 6b.

To get an insight into the achieved quality level of the gained TS-based reference point data, the average Helmert’s point error over all 594 measured points has been calculated and amounts to:

(8) $$s_{\mathrm{TS}}=2.6\;\mathrm{mm}.$$

Furthermore, to assess the quality level of the gained UAS-based PC of the chimney, based on the known (TS survey) and estimated (SfM algorithm) coordinates of the four MVPs, the average root mean square error (RMSE) has been determined and amounts to:

(9) $$s_{\mathrm{UAS}}=25.5\;\mathrm{mm}.$$

To objectively evaluate the equality of the gained accuracies of the TS-based and UAS-based data processing results, a two-tailed *F*-test has been conducted. The null hypothesis and alternative hypothesis are set:

(10) $$\begin{array}{cc} H_{0}: & s_{\mathrm{UAS}}^{2}=s_{\mathrm{TS}}^{2},\\ H_{A}: & s_{\mathrm{UAS}}^{2}\neq s_{\mathrm{TS}}^{2}. \end{array}$$

The test was conducted at the significance level of α=0.05 with the numerator freedom degrees of $f_{\mathrm{UAS}}=3$ and the denominator freedom degrees of $f_{\mathrm{TS}}=593$. Key statistical testing parameters are given in Table 3.

Results of the conducted *F*-test indicate that there is strong evidence to not accept the null hypothesis. Empirical variances, i.e., standard deviation, of the two independent experimental data sets are not equal. A difference in the accuracies of the TS-based chimney surface points and of the UAS chimney PC has been achieved, whereby the accuracy of the TS-based data set is higher by about a factor of 10. The gained accuracy assessment results are, to a certain extent, expected because the accuracy of any UAS photogrammetrically generated PC will hardly reach the level of accuracy that can be routinely obtained with a precise TS.

To draw further conclusions, the spatial accuracy of the GCPs, used to generate the chimney PC, has been evaluated and the average Helmert’s point error over all 19 GCPs has been calculated and amounts to 2.7 mm. From the gained accuracy result it can be concluded that such high spatial accuracy of the GCPs has not negatively affected the accuracy of the gained UAS-based PC. However, the number and uniform spatial distribution of GCPs is also crucial for gaining high accuracy PCs. This criterion is practically demanding to achieve in high industrial chimney UAS surveys and has not been fully achieved in this research. Furthermore, limited image resolution of the applied image acquisition camera system certainly affected the achieved lower accuracy of the UAS-based PC. However, in this study, this issue has not been individually addressed. A question arises: can such lower accuracy UAS data be enough to accurately determine the geometrical parameters of the industrial masonry chimney?

### 3.1. Cross-Sectional Modeling Results

The TS-based chimney cross section points and the UAS-based chimney PC are the main data sets for the determination of the chimney geometry, i.e., cross-sectional dimensions of the chimney and the parameters of its axis, and for the validation of the UAS photogrammetric approach in industrial chimney surveys. Prior to elliptical cross-sectional modeling of the chimney, elaborated in detail in Section 2.4.3, segmentation of the cleaned full chimney PC along the vertical (*H*-axis), with a segment height of 5 cm, was conducted. The segmentation procedure resulted in a new and reduced PC of 20,736 points, or 0.97% of the “original” full chimney PC. A visualization of the PC segmentation results is given on Figure 6c.

**Figure 7:**
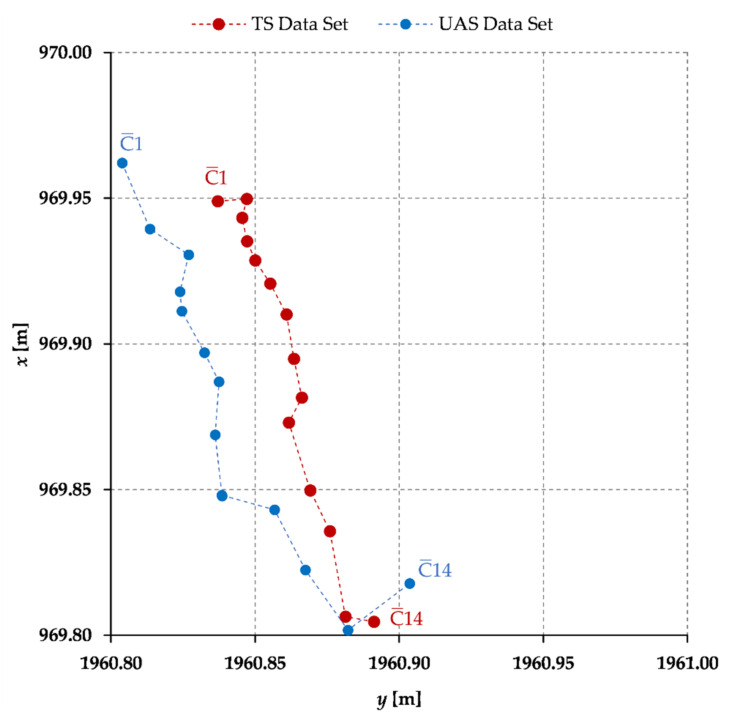
A 2D (*yx*-plane) visualization of the cross-sectional ellipse centers of the TS-based data set (red) and UAS-based data set (blue).

In total, 14 horizontal chimney cross sections, vertically spaced approx. 5 m, have been defined and the ellipse-fitting procedure was conducted on the given data, independently for the TS-based reference data and the UAS-based data. Complete results of the cross-sectional ellipse-fitting procedure are given in Table 4 (TS-based data) and Table 5 (UAS-based data). Furthermore, a 2D visualization (in the *yx*-plane) of the gained ellipse modeling results, i.e., the centers of individual chimney elliptical cross sections, is given on Figure 7.

By analyzing the gained results, a high-quality level, in both data sets, is noticeable, whereby the maximal values of standard deviations of an individual coordinate are around 3 mm. Furthermore, the standard deviation values of the UAS-based data, are constantly significantly lower, averaging at approx. 0.5 mm. This is a direct consequence of a significantly higher point number in the UAS-based mathematical ellipse-fitting procedure. The TS-based cross sections average at 42 points per cross section, while the UAS-based cross sections average at 1481 points per cross section. The standard deviation is a measure of the precision of a parameter, not its accuracy. Therefore, lower standard deviation values of UAS-based results relative to the TS-based results do not imply their higher accuracy.

By a quick visual analysis of the gained results, given on Figure 7, an inclination of the chimney structure is apparent. Results of both methods indicate the deviation of the chimney top from its base nearly 15 cm. Based on the results of cross-sectional elliptical modeling of both the TS-based data set (Table 4) and the UAS-based data set (Table 5), differences of key elliptical parameters are calculated and presented in Table 6.

**Figure 8:**
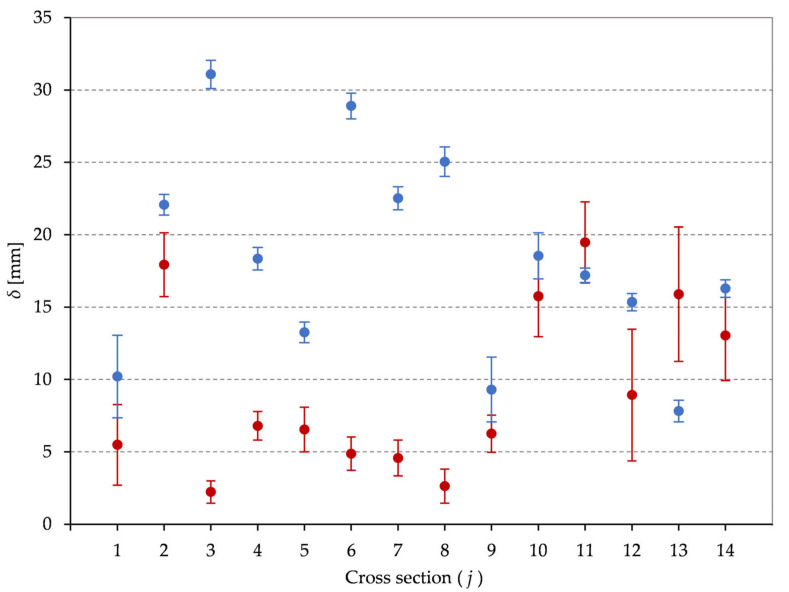
The differences between the semi-major and semi-minor ellipse axis for every cross section (*j*) with their standard deviations (error bars) for the TS-based data sets (red) and the UAS-based data sets (blue).

To investigate the cross-sectional geometry of the masonry chimney, an analysis of the semi-axis of modeled ellipses is conducted. The differences (*δ*) between the semi-major (*a*) and semi-minor (*b*) axis are calculated for every cross section in both data sets (TS- and UAS-based) with their corresponding standard deviations calculated according to error propagation [38]. The gained results are visualized on Figure 8 whereby the TS-based results are depicted in the red and the UAS-based results in the blue color.

The TS-based results are more consistent with a maximal value of $\delta_{\mathrm{TS}}^{\max}=19.5\;\mathrm{mm}$, and show a larger deviation from a circle in the upper third of the chimney (cross sections 10 to 14). This could indicate a deformation of the chimney structure which is in line with its current condition and damage (Figure 2). On the other hand, the UAS-based results are more scattered and, although better standard deviation values are gained, cannot be a basis for drawing conclusions. This is, however, not unexpected, because the deviation of the chimney structure from a circle is less than the gained accuracy of the UAS-based PC.

Furthermore, according to the results of cross-sectional elliptical modeling of the TS-based data set, the mean chimney eccentricity is determined and amounts to ${\bar{e}}_{\mathrm{TS}}=0.092$ with a standard deviation of $s_{e}^{\mathrm{TS}}=0.003$. To determine if the masonry chimney eccentricity is significantly different from zero, the Student’s *t*-test is conducted whereby the hypothesized value of the chimney eccentricity is zero, i.e., that the cross section of the chimney is a circle. The null hypothesis and alternative hypothesis are set:

(11) $$\begin{array}{cc} H_{0}: & {\bar{e}}_{\mathrm{TS}}=0,\\ H_{A}: & {\bar{e}}_{\mathrm{TS}}\neq 0. \end{array}$$

The statistical test was conducted at the significance level of 0.05 and a freedom degree of 13. Key statistical testing parameters are given in Table 7.

Results of the conducted *t*-test indicate that there is strong evidence to not accept the null hypothesis. The chimney mean eccentricity value is significantly different from zero at a confidence level of 95%. Confirming that the chimney cross-sectionally is indeed an ellipse, thus confirming our initial assumption of applying the ellipse-fitting procedure.

### 3.2. Axis Modeling Results

To evaluate the chimney axis upon the UAS-based data set, a reference axis model needs to be known in advance. To generate the reference model of the chimney axis, the TS-based data set is used, whereby an initial analysis on the optimal mathematical model to be applied is conducted. From the gained cross-sectional ellipse centers of all 14 defined cross sections within the TS-based data set, modeling of the chimney axis, according to the methodology described in Section 2.4.4., is conducted. By means of polynomial regression the chimney axis is fitted by three polynomial functions in 3D, i.e., a line, a quadratic curve, and a cubic curve. Of the three listed regression models, the optimal one in terms of the number of parameters, goodness-of-fit criteria, and model simplicity, is chosen. Therefore, higher degree functions have not been evaluated, as they would overfit the regression model. To evaluate which of the three fitted polynomial models is the optimal, three evaluation parameters, i.e., goodness-of-fit criteria, based on the residuals in the adjustment procedure have been defined:

- Weighted sum of squared residuals Ω;
- *A posteriori* standard deviation of unit weight $s_{0}$;
- Mean error distance $\bar{d}$.

The first two criteria, being well known parameters and standardized parts of the GMM, are not elaborated in detail. The mean error distance ($\bar{d}$) is the mean value of *n* Euclidian distances calculated between the *a priori* and *a posteriori* chimney cross section centers for every individual cross section in the polynomial regression procedure.

Evaluation parameters of the polynomial regression based on the reference TS data set are determined and presented in Table 8. By analyzing the gained results, a substantial increase in the goodness of the evaluation parameters of the 2nd degree polynomial with respect to the 1st degree polynomial is evident. However, further increase in the goodness of the evaluation parameters of the 3rd degree polynomial does not justify the simultaneous increase in the model’s complexity and the number of its parameters. Therefore, upon the TS-based reference data set, the quadratic curve function is evaluated as the optimal polynomial regression model for the masonry chimney axis.

After the determination of the chimney reference axis model, the validation model based on the UAS data set is generated, whereby the identical mathematical regression procedure of the 2nd degree polynomial is conducted. The final 2nd degree polynomial regression parameters of both data sets are given in Table 9.

Equal evaluation parameters of the quadratic curve model based on the validation UAS-based data set are generated and presented in Table 10. A substantial difference with respect to the evaluation parameters of the reference 2nd degree polynomial is evident. The results show that the cross-sectional centers, determined from the UAS-based data set, are more scattered and lead to the determination of the chimney axis parameters with a considerably lower accuracy compared with the reference data set.

**Figure 9:**
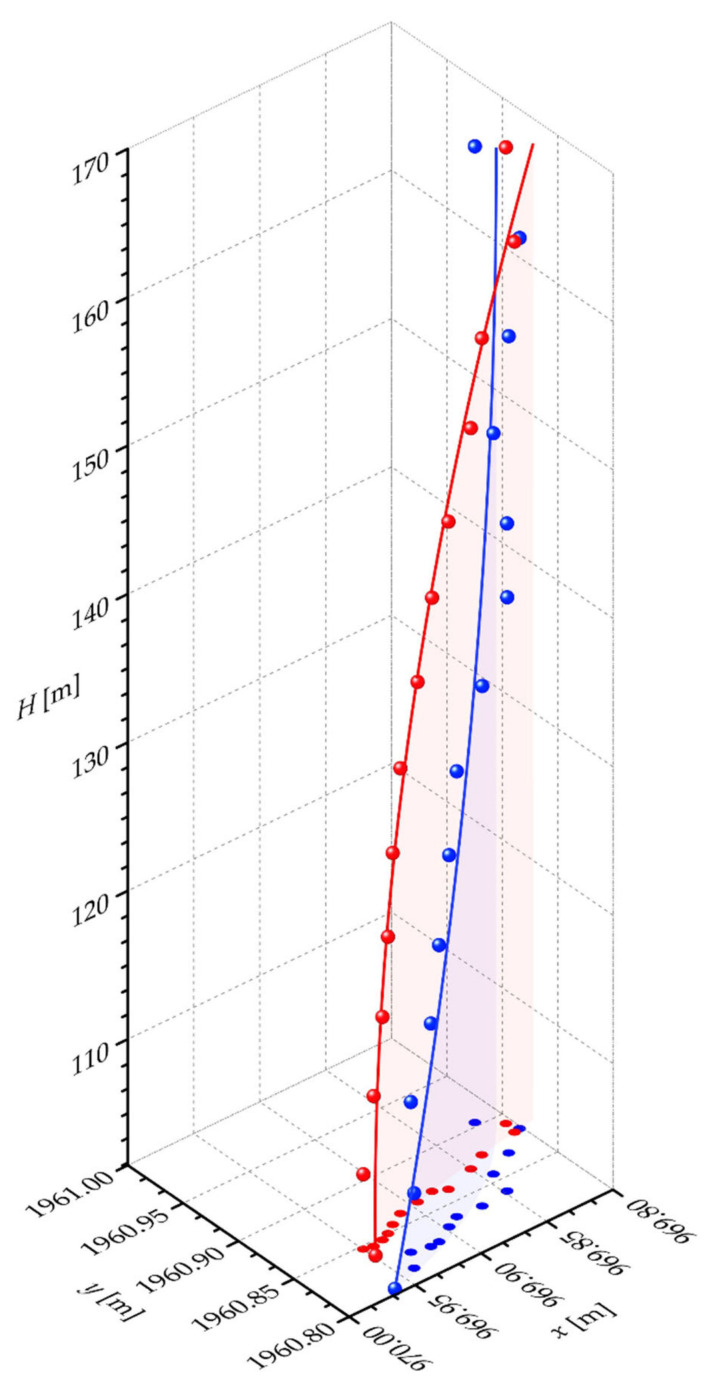
A 3D visualization of the quadratic curve and the cross-sectional ellipse centers on which the curve is determined, individually for the reference TS-based data set (red) and the validation UAS-based data set (blue).

A 3D visualization of the determined quadratic polynomial regression models, i.e., of the quadratic curves (Table 9), with the corresponding cross-sectional ellipse centers (Table 4 and Table 5) based on which the models were generated, individually for both the reference TS and the validation UAS data sets, is given on Figure 9. The TS results are depicted in red and the UAS results in blue color. Furthermore, the determined parameters of the 2nd degree polynomial regression models of both the reference and the validation data sets (Table 9) enable, the calculation of the final positions of all cross sections of the chimney as defined by the parameter value t. Results are given in Table 11 and Table 12. These coordinates represent the final chimney axis at the defined cross sections.

The final step of the chimney geometrical analysis is the determination of the vertical inclination of the chimney structure at every defined cross section. The inclination is calculated based on the unit tangent vector according to Equation (5). Furthermore, to gain an information as to whether the determined chimney inclination is significant it is compared with the maximal allowed imperfections of masonry structures as defined by the European Standard EN 1996-1-1:2005. Results on the chimney inclination evaluation are given in Table 13.

The maximal inclination values decrease with the increase in the structure’s height. This is fully logical because higher masonry structures cannot physically sustain equal inclinations as lower structures would. The results of the determined referent geometry of the chimney based on the TS data set, i.e., the referent inclination values, show that the chimney inclination at the top is five time larger than at the bottom whereby the maximal inclination value is already surpassed at approx. 24.3 m of chimney height. More than half of the chimney does not, regarding its inclination, comply with the guidelines of the European Standard EN 1996-1-1:2005.

Further analysis of the chimney inclination results generated by the UAS survey showed a great disagreement with the reference inclination values. Different orientations of the quadratic functions were gained. While the reference chimney axis is concave downward, the validation chimney axis is concave upward. This is also evident from the sign of the quadratic polynomial function coefficients of both models. Based on all experimental results, it cannot be concluded that the UAS-based PC data of lower accuracy with respect to the TS-based data, can be used to determine the geometrical parameters of industrial masonry chimneys.

## 4. Conclusions

This paper has investigated the application of two independent methods for the determination of structural geometrical parameters of an industrial masonry chimney. While various modern geodetic measurement techniques have been proven as applicable for this task, the application of UAS photogrammetry has not yet been fully investigated. Therefore, in this research the UAS photogrammetric approach has been evaluated and thus gained results were compared with referent values determined by a TS-based survey. Key structural geometrical parameters of a masonry chimney addressed in this research are its axis inclination with respect to the vertical and its cross-sectional geometry. Inclination of the chimney body is essential from its stability analysis point of view [1]. Methodologically, targeted geometrical parameters have been determined based on a double modeling procedure. Cross-sectional chimney geometry has been determined by an elliptical regression method and the inclination of its axis by subsequent polynomial regression, thereby the weighted least-squares method has been applied. The chimney axis inclination has been evaluated according to guidelines of the European Standard EN 1996-1-1:2005 [27].

As a reference frame for all conducted measurements and calculation, a precise and high quality GRN has been established around the industrial masonry chimney. Experimental results of TS and UAS field data acquisition and processing confirm our initial assumption regarding the overall accuracy of both acquired data sets. The achieved average accuracy of the UAS photogrammetrically generated PC is significantly lower than the achieved average accuracy of the TS data set, concretely, by a factor of 10. Unfavorable spatial distribution of the GCPs in our research, whereby the position of GCPs has been defined by characteristic natural features on the chimney surface, is the main reason for the achieved significant accuracy difference. Generally, all results based on the TS data set, regarding the structural geometrical parameters of the chimney, show consistently higher relative and absolute accuracy compared with the results of the UAS data processing. The determined results on chimney referent cross-sectional geometry, based on the TS data set, confirm our initial assumption on the ellipticity of the chimney structure. Furthermore, they indicate deformations of the upper third of the chimney may exist. However, based to the UAS validation results the same conclusion cannot be given. Results are more scattered and no correlation with referent values can be determined. This conclusion is in line with the achieved accuracy difference of both data sets, because the determined chimney deformation is smaller than the achieved accuracy of the UAS-generated PC. Our experimental results on the referent axis determination showed that the 3D quadratic curve model is an optimal representation of the spatial chimney axis whereby a mean error distance of the axis model of 4.3 mm has been achieved. However, the validation axis model, based on the UAS data set, resulted in the mean error distance of 9.6 mm, showing a considerably lower accuracy of the generated model. The chimney axis inclination determination and analysis results, based on the referent TS data set, showed that a concerning axis inclination exists, whereby approx. only the bottom 24.3 m of the chimney structure fulfills the requirements of the European Standard. Results on axis inclination determination based on the validation UAS data set are not fully in agreement with the referent results.

In conclusion, in our research we have successfully applied two independent methods, i.e., the TS and UAS photogrammetric surveys, of industrial masonry chimney structural geometrical parameters determination, and a comparison of the gained results is presented in this paper. However, based on our experimental results, the applicability of the UAS photogrammetric survey needs further research. A clear conclusion as to whether this method can be applied to determine accurate values of the chimney geometry parameters cannot be given. Our results indicate that much attention must be given to the quality and accuracy of the photogrammetrically generated PC, as it is the foundation for further calculations. In future research more attention to the number and uniform spatial distribution of GCPs is proposed whereby more quality image acquisition camera systems could be applied.

A few potential limitations of the conducted study need to be considered. Firstly, the present study has investigated the masonry chimney strictly from a static point of view wherein only its geometrical parameters where in focus. An analysis of the chimney as a dynamic (cause-response) system requires a comprehensive interdisciplinary approach. Therefore, one limitation regarding the fact that the TS and UAS photogrammetric surveys of the chimney have been conducted at different dates and hence relate to different chimney spatial states, needs to be considered. However, the analyzed chimney is more than 90 years old, of which for nearly 30 years it was not in use. Hence, a long periodical deformation of the chimney may exist and be of a small amount. Therefore, it is reasonable to assume that a month and a half is not a significant time frame regarding such long periodical deformations and that modelling of the chimney’s dynamics, in that aspect, can be neglected. Moreover, atmospheric conditions during the two field surveys (20 May 2019, and 5 July 2019) were equal and without wind. Additionally, during the time between surveys, no earthquakes were recorded in the wider area. Therefore, it is reasonable to conclude that the change of the spatial state of the chimney can be neglected and that the two independent surveys refer to the same chimney spatial axis. Secondly, within this research, prior to the chimney PC segmentation process, manual cleaning of the PC was conducted whereby all outlier points (i.e., PC noise) that do not relate to the chimney surface have been removed. Such an approach is certainly operator dependent and time-consuming. In further research, a more objective, numerical, and mathematically assisted procedure is advised, e.g., the RANSAC approach [58].

Finally, based on the gained results and the detected structural deformations of the researched industrial masonry chimney of the former Duga Resa Cotton Industry, to fully assess its structural stability, further research from a civil engineering point of reference is needed. It can only be emphasized that dangerous permanent deformations are plausible.

## Figures and Tables

**Table 1:** Summary of field data acquisition.

Survey	Date of Data Acquisition	Sensor
GRN ^1^ survey	7 May 2019	Leica Geosystems TCRP1201+ R400
Chimney cross-sectional survey	20 May 2019	Leica Geosystems TCRP1201+ R400
GCP ^2^ and MVP ^3^ survey	20 May 2019	Leica Geosystems TCRP1201+ R400
UAS ^4^ image acquisition	5 July 2019	DJI Phantom 4 Pro

^1^ GRN—Geodetic Reference Network; ^2^ GCP—Ground Control Point; ^3^ MVP—Model Validation Point; ^4^ UAS—Unmanned Aerial System.

**Table 2:** Network adjustment results with corresponding quality criteria parameters: standard deviations of the coordinates $\left(s_{y},s_{x},s_{H}\right)$; and semi-axes of the 95% absolute confidence error ellipsoids $\left(a,b,c\right)$.

Point	$\begin{array}{c} y\\ \left[m\right] \end{array}$	$\begin{array}{c} x\\ \left[m\right] \end{array}$	$\begin{array}{c} H\\ \left[m\right] \end{array}$	$\begin{array}{c} s_{y}\\ \left[mm\right] \end{array}$	$\begin{array}{c} s_{x}\\ \left[mm\right] \end{array}$	$\begin{array}{c} s_{H}\\ \left[mm\right] \end{array}$	$\begin{array}{c} a\\ \left[mm\right] \end{array}$	$\begin{array}{c} b\\ \left[mm\right] \end{array}$	$\begin{array}{c} c\\ \left[mm\right] \end{array}$
P1 *	2000.001	1000.000	100.001	0.3	0.2	0.2	1.1	1.0	0.8
P2 *	2000.000	941.078	100.165	0.2	0.3	0.2	1.0	0.9	0.9
P3 *	1972.123	996.362	99.777	0.2	0.2	0.2	1.0	0.8	0.8
P4 *	1958.200	941.211	99.661	0.2	0.2	0.2	1.1	0.9	0.8
P5	1903.271	944.929	99.181	0.5	0.6	0.4	2.3	2.0	1.6
P6	1921.404	998.888	99.235	0.6	0.5	0.4	2.7	1.9	1.7

* Geodetic Reference Network (GRN) datum points.

**Table 3:** Key parameters of the conducted two-tailed *F*-test of the equality of variances.

Test Statistics F	Lower Critical Value $F_{1-\alpha /2}$	Upper Critical Value $F_{\alpha /2}$	Hypothesis Accepted
98.49	0.07	3.14	$H_{A}$

**Table 4:** Results of TS-based chimney elliptical cross-sectional modeling.

Cross Section	$\begin{array}{c} y\\ \left[m\right] \end{array}$	$\begin{array}{c} x\\ \left[m\right] \end{array}$	$\begin{array}{c} H\\ \left[m\right] \end{array}$	$\begin{array}{c} s_{y}\\ \left[mm\right] \end{array}$	$\begin{array}{c} s_{x}\\ \left[mm\right] \end{array}$	$\begin{array}{c} s_{H}\\ \left[mm\right] \end{array}$	$\begin{array}{c} a\\ \left[m\right] \end{array}$	$\begin{array}{c} s_{a}\\ \left[mm\right] \end{array}$	$\begin{array}{c} b\\ \left[m\right] \end{array}$	$\begin{array}{c} s_{b}\\ \left[mm\right] \end{array}$
$\bar{C}1$	1960.837	969.949	101.557	1.6	1.5	1.1	2.770	2.2	2.764	1.7
$\bar{C}2$	1960.847	969.950	106.544	2.0	0.7	2.7	2.695	1.0	2.677	2.0
$\bar{C}3$	1960.846	969.943	111.623	0.6	0.5	2.1	2.536	0.4	2.534	0.7
$\bar{C}4$	1960.847	969.935	116.525	0.8	0.6	1.9	2.399	0.6	2.392	0.8
$\bar{C}5$	1960.850	969.929	121.497	1.4	0.8	1.2	2.305	1.2	2.298	1.0
$\bar{C}6$	1960.855	969.921	126.545	1.0	0.6	2.2	2.211	0.8	2.206	0.8
$\bar{C}7$	1960.861	969.910	131.506	1.0	0.6	2.0	2.121	0.7	2.116	1.0
$\bar{C}8$	1960.863	969.895	136.544	0.7	0.6	2.7	2.032	0.5	2.029	1.1
$\bar{C}9$	1960.866	969.882	141.504	0.8	0.7	2.8	1.947	1.1	1.940	0.7
$\bar{C}10$	1960.862	969.873	146.494	1.1	2.5	1.9	1.853	1.2	1.837	2.6
$\bar{C}11$	1960.869	969.850	151.423	1.2	1.2	3.4	1.773	1.4	1.754	2.5
$\bar{C}12$	1960.876	969.836	156.538	1.6	2.5	2.4	1.672	1.4	1.663	4.3
$\bar{C}13$	1960.881	969.806	161.517	1.5	2.1	1.5	1.591	4.5	1.575	1.3
$\bar{C}14$	1960.891	969.805	167.290	1.8	1.9	3.1	1.511	2.4	1.498	1.9

**Table 5:** Results of UAS-based chimney elliptical cross-sectional modeling.

Cross Section	$\begin{array}{c} y\\ \left[m\right] \end{array}$	$\begin{array}{c} x\\ \left[m\right] \end{array}$	$\begin{array}{c} H\\ \left[m\right] \end{array}$	$\begin{array}{c} s_{y}\\ \left[mm\right] \end{array}$	$\begin{array}{c} s_{x}\\ \left[mm\right] \end{array}$	$\begin{array}{c} s_{H}\\ \left[mm\right] \end{array}$	$\begin{array}{c} a\\ \left[m\right] \end{array}$	$\begin{array}{c} s_{a}\\ \left[mm\right] \end{array}$	$\begin{array}{c} b\\ \left[m\right] \end{array}$	$\begin{array}{c} s_{b}\\ \left[mm\right] \end{array}$
$\bar{C}1$	1960.804	969.962	101.557	3.0	0.8	0.4	2.793	2.2	2.783	1.8
$\bar{C}2$	1960.814	969.939	106.544	0.5	0.3	0.4	2.713	0.4	2.691	0.6
$\bar{C}3$	1960.827	969.931	111.623	0.4	0.6	0.3	2.556	0.8	2.525	0.6
$\bar{C}4$	1960.824	969.918	116.525	0.4	0.5	0.3	2.413	0.6	2.394	0.4
$\bar{C}5$	1960.825	969.911	121.497	0.3	0.4	0.3	2.313	0.6	2.300	0.4
$\bar{C}6$	1960.832	969.897	126.545	0.4	0.5	0.3	2.227	0.8	2.198	0.5
$\bar{C}7$	1960.837	969.887	131.506	0.4	0.5	0.3	2.132	0.7	2.109	0.4
$\bar{C}8$	1960.836	969.869	136.544	0.4	0.6	0.4	2.045	0.9	2.020	0.6
$\bar{C}9$	1960.839	969.848	141.504	0.9	1.9	0.5	1.955	2.1	1.946	0.7
$\bar{C}10$	1960.838	969.848	146.494	1.0	1.2	0.5	1.865	1.5	1.846	0.6
$\bar{C}11$	1960.857	969.843	151.423	0.3	0.3	0.4	1.776	0.4	1.759	0.3
$\bar{C}12$	1960.867	969.822	156.538	0.3	0.4	0.4	1.681	0.5	1.666	0.3
$\bar{C}13$	1960.882	969.802	161.517	0.4	0.5	0.5	1.587	0.7	1.580	0.3
$\bar{C}14$	1960.904	969.818	167.290	0.3	0.4	0.4	1.515	0.5	1.499	0.4

**Table 6:** Differences of ellipse modeling results between the TS- and the UAS-based data sets.

Cross Section	$\begin{array}{c} \Delta y\\ \left[m\right] \end{array}$	$\begin{array}{c} \Delta x\\ \left[m\right] \end{array}$	$\begin{array}{c} \Delta H\\ \left[m\right] \end{array}$	$\begin{array}{c} \Delta a\\ \left[m\right] \end{array}$	$\begin{array}{c} \Delta b\\ \left[m\right] \end{array}$
$\bar{C}1$	−0.033	0.013	0.000	0.023	0.018
$\bar{C}2$	−0.034	−0.010	0.000	0.018	0.014
$\bar{C}3$	−0.019	−0.013	0.000	0.020	−0.009
$\bar{C}4$	−0.023	−0.017	0.000	0.014	0.002
$\bar{C}5$	−0.025	−0.017	0.000	0.008	0.001
$\bar{C}6$	−0.023	−0.024	0.000	0.016	−0.008
$\bar{C}7$	−0.023	−0.023	0.000	0.011	−0.007
$\bar{C}8$	−0.027	−0.026	0.000	0.013	−0.010
$\bar{C}9$	−0.028	−0.034	0.000	0.009	0.006
$\bar{C}10$	−0.023	−0.025	0.000	0.012	0.009
$\bar{C}11$	−0.012	−0.007	0.000	0.003	0.006
$\bar{C}12$	−0.009	−0.013	0.000	0.010	0.003
$\bar{C}13$	0.001	−0.005	0.000	−0.004	0.004
$\bar{C}14$	0.012	0.013	0.000	0.004	0.001

**Table 7:** Key parameters of the Student’s *t*-test for the significance of the chimney eccentricity.

Test Statistic T	Critical Region $T_{1-\alpha /2}$	Hypothesis Accepted
26.34	$\left.\langle -\infty ,-2.160\right]\cup \left[2.160,\infty \rangle \right.$	$H_{A}$

**Table 8:** Description and evaluation parameters of the polynomial regression models of the chimney axis based on the reference TS data set.

Regression Model	Polynomial Degree	Number of Parameters	$\begin{array}{c} \Omega \\ \left[mm^{2}\right] \end{array}$	$\begin{array}{c} s_{0}\\ \left[mm\right] \end{array}$	$\begin{array}{c} \bar{d}\\ \left[mm\right] \end{array}$
Line	1	3	3068.22	11.3	15.3
Quadratic cure	2	6	236.83	2.7	4.3
Cubic curve	3	9	161.45	2.3	3.7

**Table 9:** The 2nd degree polynomial function (quadratic curve) parameters of both the reference TS-based data set and of the validation UAS-based data set.

Polynomial Function Parameter	TS Data Set		UAS Data Set	
	Value [m]	St. Deviation [m]	Value [m]	St. Deviation [m]
${\bar{a}}_{y}$	0.0007260	0.0000757	0.0007143	0.0001636
${\bar{a}}_{x}$	−0.0004294	0.0000612	−0.0029607	0.0002196
${\bar{a}}_{H}$	1.0000005	0.0001465	0.9999953	0.0001563
${\bar{b}}_{y}$	−0.0000002	0.0000017	0.0000101	0.0000030
${\bar{b}}_{x}$	−0.0000302	0.0000015	0.0000097	0.0000040
${\bar{b}}_{H}$	−0.0000001	0.0000028	0.0000000	0.0000031

**Table 10:** Evaluation parameters of the 2nd degree regression model of the chimney axis based on the validation UAS-based data set.

Regression Model	Polynomial Degree	Number of Parameters	$\begin{array}{c} \Omega \\ \left[mm^{2}\right] \end{array}$	$\begin{array}{c} s_{0}\\ \left[mm\right] \end{array}$	$\begin{array}{c} \bar{d}\\ \left[mm\right] \end{array}$
Quadratic cure	2	6	7947.85	15.5	9.6

**Table 11:** Final regression results based on the reference TS data set; parameter *t* defines the position of the cross section along the quadratic cure and determines its 3D coordinates.

Cross Section	$\begin{array}{c} t\\ \left[1\right] \end{array}$	$\begin{array}{c} y\\ \left[m\right] \end{array}$	$\begin{array}{c} x\\ \left[m\right] \end{array}$	$\begin{array}{c} H\\ \left[m\right] \end{array}$	$\begin{array}{c} s_{y}\\ \left[mm\right] \end{array}$	$\begin{array}{c} s_{x}\\ \left[mm\right] \end{array}$	$\begin{array}{c} s_{H}\\ \left[mm\right] \end{array}$
$\hat{C}1$	0.00000	1960.837	969.949	101.557	1.6	1.5	1.1
$\hat{C}2$	4.98701	1960.841	969.946	106.544	0.3	0.3	0.7
$\hat{C}3$	10.06601	1960.844	969.942	111.623	0.6	0.5	1.2
$\hat{C}4$	14.96801	1960.848	969.936	116.525	0.8	0.6	1.6
$\hat{C}5$	19.94001	1960.851	969.928	121.497	0.9	0.7	1.9
$\hat{C}6$	24.98802	1960.855	969.919	126.545	1.0	0.7	2.0
$\hat{C}7$	29.94903	1960.859	969.909	131.506	1.0	0.7	2.1
$\hat{C}8$	34.98705	1960.862	969.897	136.544	0.9	0.7	2.0
$\hat{C}9$	39.94707	1960.866	969.884	141.504	0.9	0.8	2.0
$\hat{C}10$	44.93707	1960.869	969.869	146.494	1.0	1.1	1.9
$\hat{C}11$	49.86611	1960.873	969.852	151.423	1.2	1.4	2.0
$\hat{C}12$	54.98113	1960.876	969.834	156.538	1.6	1.8	2.4
$\hat{C}13$	59.96019	1960.880	969.815	161.517	2.1	2.4	3.0
$\hat{C}14$	65.73318	1960.884	969.790	167.290	2.8	3.1	4.0

**Table 12:** Final regression results based on the validation UAS data set; parameter *t* defines the position of the cross section along the quadratic cure and determines its 3D coordinates.

Cross Section	$\begin{array}{c} t\\ \left[1\right] \end{array}$	$\begin{array}{c} y\\ \left[m\right] \end{array}$	$\begin{array}{c} x\\ \left[m\right] \end{array}$	$\begin{array}{c} H\\ \left[m\right] \end{array}$	$\begin{array}{c} s_{y}\\ \left[mm\right] \end{array}$	$\begin{array}{c} s_{x}\\ \left[mm\right] \end{array}$	$\begin{array}{c} s_{H}\\ \left[mm\right] \end{array}$
$\hat{C}1$	0.00000	1960.804	969.962	101.557	3.0	0.8	0.4
$\hat{C}2$	4.98682	1960.808	969.948	106.544	0.7	1.0	0.7
$\hat{C}3$	10.06546	1960.812	969.933	111.623	1.4	1.8	1.3
$\hat{C}4$	14.96695	1960.817	969.920	116.525	1.8	2.4	1.7
$\hat{C}5$	19.93996	1960.822	969.907	121.497	2.1	2.9	2.0
$\hat{C}6$	24.98773	1960.828	969.894	126.545	2.3	3.1	2.1
$\hat{C}7$	29.94798	1960.834	969.882	131.506	2.4	3.2	2.2
$\hat{C}8$	34.98676	1960.841	969.870	136.544	2.4	3.1	2.2
$\hat{C}9$	39.94628	1960.849	969.859	141.504	2.2	2.9	2.1
$\hat{C}10$	44.93727	1960.856	969.849	146.494	2.1	2.7	2.1
$\hat{C}11$	49.86580	1960.865	969.839	151.423	2.0	2.5	2.2
$\hat{C}12$	54.98008	1960.874	969.829	156.538	2.1	2.6	2.7
$\hat{C}13$	59.95902	1960.883	969.819	161.517	2.6	3.2	3.4
$\hat{C}14$	65.73205	1960.894	969.809	167.290	3.5	4.4	4.5

**Table 13:** Results on chimney inclination evaluation for both the referent TS-based and the validation UAS-based data sets.

Cross Section	$\begin{array}{c} \varphi_{\mathrm{TS}}\\ \left[{{}^{\circ}}^{\prime \prime \prime }\right] \end{array}$	$\begin{array}{c} \varphi_{\mathrm{UAS}}\\ \left[{{}^{\circ}}^{\prime \prime \prime }\right] \end{array}$	$\begin{array}{c} \varphi_{\max}\\ \left[{{}^{\circ}}^{\prime \prime \prime }\right] \end{array}$
$\hat{C}1$	0°2′54″	0°10′28″	/
$\hat{C}2$	0°3′32″	0°10′14″	0°15′24″
$\hat{C}3$	0°4′21″	0°10′01″	0°10′50″
$\hat{C}4$	0°5′13″	0°9′50″	0°8′53″
$\hat{C}5$	0°6′08″	0°9′39″	0°7′42″
$\hat{C}6$	0°7′07″	0°9′30″	0°6′53″
$\hat{C}7$	0°8′05″	0°9′22″	0°6′17″
$\hat{C}8$	0°9′05″	0°9′15″	0°5′49″
$\hat{C}9$	0°10′05″	0°9′10″	0°5′26″
$\hat{C}10$	0°11′05″	0°9′06″	0°5′08″
$\hat{C}11$	0°12′05″	0°9′04″	0°4′52″
$\hat{C}12$	0°13′08″	0°9′03″	0°4′38″
$\hat{C}13$	0°14′09″	0°9′04″	0°4′26″
$\hat{C}14$	0°15′20″	0°9′07″	0°4′14″

## Data Availability

The data presented in this study are available on request from the corresponding author. The data are not publicly available due to the conditions and requirements of the Regulation on Aerial Photography of The Government of the Republic of Croatia.
